# A Plea for Life Assurance

**Published:** 1878-01

**Authors:** Jas. B. Hodgkin


AMERICAN JOURNAL

OF

DENTAL SCIENCE.

Vol. XI. THIRD SERIES—JANUARY, 1878. No. 9.

### ARTICLE I.

### *A Plea for Life Assurance.*

Read before the Virginia State Dental Association at its Annual Meeting in Richmond, December 11th, 1877.

Why it is so, is a matter not properly within the scope of this paper to discuss, but all who read this will agree with me in the statement that dentists save but a small percentage of their gross earnings, compared with most other professions or occupations. The number of those who die—and we are a short-lived class, dropping into early graves often, and rarely prolonging life to old age—possessed of even moderate fortunes to bequeath to those who come after them, is very small; while the far greater proportion either accumulate little, or make only a comfortable living, or, worse still become, as sight and nerves fail, absolutely poor. It would seem as though we ought, with fair fees and a moderate practice, to accumulate something of a competency, for the conviction which obtains almost universally among our patients that the dentists handle a great deal of money, must have some foundation in fact. Dr. Robert Arthur, in his cleverly written and as yet unanswered book on "Decay of the Teeth," in giving a summary of the present status of dentistry, shows why it is not likely that the honest practitioner is likely to do well financially; but our patients seem to feel in these hard times, as they pay the bills, that they are, to use the words of a medical friend of mine, "swingeing bills" indeed. But for all that, somehow or other the fees, though seemingly fair in the aggregate, do not amount to much by the time the butcher and baker, the landlord, &c., are paid, and the depot man is settled with for the foil and other necessary means of life and of work. However much we may be sanguine in youth of reaping a rich harvest out of so apparently promising a field as dentistry, most of us who have reached middle life lose the vividness of that dream of fortune which was so entrancing in earlier days, and many realize that a competency, or a comfortable living, is all they may reasonably hope for.

Yet the desire to do something by which our little ones may be in some measure provided for when we cease to be, is among the strongest and the noblest of our impulses; for who wishes to leave his posterity to battle with adversity in a selfish world, unprovided with almost the only thing the world recognises as strong for defence! We feel the burden of this as the gravest and at the same time one of the sweetest of the cares that engross us, that when the hands that are now toiling are folded in death and their restless endeavor forever stilled, we shall by their toil leave behind that which shall place our dear ones beyond pinching want or mendicancy.

And thus it comes that almost all of us are holding policies in some one or the other of the numerous Life Assurance Companies which are established all over our land, and whose agents and representatives are found in almost every corner of the country, urging the claims of their respective rival corporations. And this is almost the only hope of thousands now, and this list includes many dentists in these truly hard times, when we feel that we cannot push our patients with big bills, and *must* work at what they call "reasonable figures,"—we put our surplus cash in some such investment, with the noble purpose in view of insuring our lives for the benefit of our wives and offspring.

I am sure that for a long time no institutions attained such popularity as Life Assurance Companies. It seems so simple a matter, the calculations so readily made, the conclusions so reliable, that few men who were approached on the subject had the hardihood to question the statements as to the certainty of the profits sure to arise from such investments. And so they became, as I said, the most amazingly popular of institutions; and I well remember with what a sense of pride I invested almost my first earnings—certainly my first surplus ones—in a policy, the company issuing which was represented, and I doubt not innocently enough by the gentleman who was its efficient agent, and a personal friend of mine, as "Safe Sir! perfectly safe I assure you." And I will recall how comfortable I felt that my wife (there were no children then) was to get the $5,000 in case of my unlucky decease. But alas! after paying into the hands of this treacherous corporation some six or seven hundred dollars, my magnificient company collapsed, and the pretentious piece of parchment they issued as a policy, proved as worthless on my hands as the confederate notes they used to pay us poor soldiers in the land and days of "Dixie."

I doubt if there is a man outside of the ring of the managers of these companies themselves, now holding a policy of life assurance who feels perfectly safe, the record of failures is so startling. I have the authority of a prominent Insurance Journal for the statement that no less than fifteen of these institutions in the United States are now in the hands of receivers; and we all know what that means, for who ever got anything from a receiver? Failure after failure has occurred, until the confidence of the public in such institutions is utterly shaken, and indeed it may well be. A late writer quoting the aphorism that "figures can't lie," adds that "notwithstanding this inability on the part of the integers, they are nevertheless the most active and nimble little liars in existence;" and I may add to this that one need only to listen to the figures quoted by a life assurance agent to feel that the ability of the figures to be manipulated is immense indeed.

I have before me a pamphlet containing statistics of the Life Assurance Companies that have been and are still in existence in this country, and the list is a startling one indeed as evidence of the utter want of stability in institutions and corporations, the representatives of which would lead us to believe are the most stable and safe of all deposits for money. The total number of companies that have existed in the United States since life assurance was instituted, is 205. The total number of failures of these companies is 144. Sixty nine per cent. of the whole number have failed!

The average age of these companies is 10 years. Of the solvent companies, their average age is 17 years; of the bankrupt companies 6 years. The bankrupt companies had liabilities of nearly forty-seven millions of dollars.

A man more familiar with the subject under discussion than any other probably in the United States, says in this connection:

"It is a startling fact that, in round numbers, nine policies lapse by forfeiture and surrender when one is terminated by death; also that the average duration or lifetime of a policy is about seven years only."

And it is worth while to state that it is probable that about thirty-five millions of dollars are paid annually in this country for life assurance, while according to the above authority, only one-tenth of this sum is paid out to policy holders.

But I must not weary you with these details, or burden you with statistics. Enough is said to show that the methods of life assurance as managed in this country are radically defective, and that hereafter the prudent man will hesitate before trusting to such precarious means of leaving an inheritance to his family.

I may be allowed here the relation of a personal incident in connection with this subject which is so illustrative of the fatal defects of the whole system as to be worth placing on record. Sitting only a few days ago in the back office of one of the most popular and (so considered) safest of these companies, I was entertained whilst waiting for the return of the manager, by one of the persons interested in the office management with a relation of some of the economical ways in which the affairs of the company were conducted. He cited the fact that the laws of the State of Virginia compelled a company organized outside of the State to make a deposit with the State Treasurer of $50,000, as a security to policy holders in that State; and chuckled over the fact that they had for $12,000, purchased Virginia State bonds, representing the sum required by law, and deposited these. In other words the security the citizens of Virginia have against fraud is not $50,000, but $12,000. You will see by this how nominal is the forfeiture in case the affairs of the company are badly managed, or more properly, rascally managed, for that is indeed rascality which would disappoint the hopes of the widow and orphan. However, the late action of a righteous judge in New York, in sentencing one of these gentlemen to the State's prison for five years for swearing to a false statement of the condition of his company, may have some influence in the right direction. Still, in Virginia no such redress could be had in the case of companies located outside of the State.

And yet we feel that some means ought to be devised—at least we would cordially hail some means, less costly in outlay, and more thoroughly mutual, and safer than those whose sole aim seems self-aggrandisement, and I am sure that a scheme which has proven safe and cheap would be welcomed.

I call your attention to such a method of Life Assurance—a plan in such successful operation as to make it no longer an unsolved problem. In the city of Washington, (I take this location because rather more familiar to us all) exists an organization called the "Masonic Mutual Relief Association of the District of Columbia." In this society are admitted masons in good standing, who can pass an examination by the physician appointed for the purpose. The initiation fee is small, I think only $2. On the death of any member of the society an assessment is made of one dollar and ten cents on each survivor, the dollar being paid by the proper officer over to the family of the deceased, the smaller sum going to defray current expenses. Any member failing to pay the assessment, is, after suitable notice, dropped from the rolls.

This is in brief the plan of relief, and its working is so admirable as to prove it a solid success. One year from its organization the society numbered 170 members; in another year it had increased to 351. At present it numbers about 1500, which is the limit of its charter. In these nine years the society has paid out about $155,000. Last year it paid out about $33,000, and received about $34,000, the aim being not to accumulate a large surplus fund, only about $5,500 having gathered so far. It has lost by non-payment of assessments about $4,000.

There are similar Masonic Associations all over the country, charging an initiation fee of from $2 to $10, with graded rates for the various ages of the applicants. You are probably, some of you, familiar with a similar organization in existence among the Episcopal Clergymen of your State, the members paying a regular assessment on the death of a member. It is a deservedly popular institution.

Now, in view of the fact that our faith in the popular methods of Life Assurance is so shaken of late: in consequence of the facts given above of this untrustworthiness, and in view of the success of this plan of Mutual Associations as seen among the Masonic fraternity, and others, could not such an organization be perfected among the Dentists, and thus make the members of our profession not only more mutually interested in each other, but be the means of support to our children in the event of our death? Some simple plan of assessment, with an incorporated association, and a bonded treasurer, (and a very little simple machinery would answer,) and thus be the means of relieving our minds of the anxiety and distress which must be ours in the contemplation of the uncertainty of our being able to leave material wealth behind us.

What I propose is that an association be formed, called the "Mutual Relief Association of Alumni of the Baltimore College of Dental Surgery," understanding of course that it is open to alumni of all colleges, and to all dentists in good standing, who can pass a medical examination, such as he would be subjected to by the rules of any ordinary assurance company. I have not perfected nor indeed outlined any plan for this organization, but only present the idea and ask for hints and suggestions, and your cordial co-operation. As fellow-workers in a common cause, we should feel a common sympathy in all that pertains to each other's welfare; we should be interested that the wants of one are the wants of all, and that some system of mutual help and protection should be established among us. Why, I know not, but there seems to be too much of a repelling element among us, as though we were similarly electrified; there is too little of fraternal intercourse, too little of harmony, of *esprit de corps* even, and I cannot help feeling that an organization having for its aim mutual relief in that great calamity which must come to all, should be entitled to cordial appreciation and approval.

A committee was appointed at the last Annual Meeting of the Alumni Association of the Baltimore College of Dental Surgery in March last, to whom was committed the elaboration of a plan of Life Assurance for the use of this Association. It will report at the next meeting of the Association, when I trust that it will be seen that the Dental Profession is not slow to appreciate the advantages of such mutual organizations, and that from every quarter the names will come in for membership.

James B. Hodgkin.

